# The Law in General Practice

**Published:** 1908-12

**Authors:** 


**The Law in General Practice.** By STANLEY B. ATKINSON, M.A., M.B., B.Sc. Pp. viii., 239. London: Henry Frowde and Hodder and Stoughton. 1908.

Mr. Atkinson is well qualified to write upon this subject, and we can thoroughly recommend his book to all medical men commencing, or not fully experienced, in practice. It deals with a large number of contingencies in matters forensic, ethical and medical, and abounds with useful hints and profitable warnings. The book is easily readable, and, if well digested, should prove of the utmost value to the young professional man, in affording him a ready-made experience and helpful counsel in dealing with many perplexing occasions almost certain to beset his early career.

